# Oral exposure to high concentrations of polystyrene microplastics alters the intestinal environment and metabolic outcomes in mice

**DOI:** 10.3389/fimmu.2024.1407936

**Published:** 2024-11-12

**Authors:** Yuka Hasegawa, Takuro Okamura, Yuriko Ono, Takahiro Ichikawa, Yuto Saijo, Naoko Nakanishi, Ryoichi Sasano, Masahide Hamaguchi, Hirohisa Takano, Michiaki Fukui

**Affiliations:** ^1^ Department of Endocrinology and Metabolism, Graduate School of Medical Science, Kyoto Prefectural University of Medicine, Kyoto, Japan; ^2^ AiSTI Science Co., Ltd., Wakayama, Japan; ^3^ Graduate School of Global Environmental Studies, Kyoto University, Kyoto, Japan; ^4^ Institute for International Academic Research, Kyoto University of Advanced Science, Kyoto, Japan

**Keywords:** toxicology, environmental science, microplastics, inflammation, dysbiosis

## Abstract

**Introduction:**

Oral exposure to microplastics (MPs) is a global health concern. In our previous study, MPs induced glucose intolerance and non-alcoholic fatty liver disease (NAFLD) under a high-fat diet-induced leaky gut syndrome (LGS). This study aims to evaluate the effects of high concentrations of MP on lipid metabolism under normal dietary conditions and to assess the changes in the intestinal tract resulting from MP exposure.

**Methods:**

C57BL6/J mice were fed a normal diet (ND) without polystyrene MPs (PS-MPs) or with PS-MPs (1000 µg/L or 5000 µg/L) for six weeks. Subsequently, intestinal permeability, gut microbiota, and metabolite levels in the serum, feces, and liver were determined.

**Results:**

Mice fed the ND showed no increase in intestinal permeability in either group. However, high MPs concentrations led to increased serum lipid levels and exacerbated fatty liver function. Oral exposure to MPs did not affect the number of innate lymphoid cells or short-chain fatty acids in the intestine. However, it increased the number of natural killer cells, altered the gut microbiota, induced inflammation, and modulated the expression of genes related to nutrient transport in the intestine. The severity of intestinal disturbance tended to worsen with dose.

**Discussion:**

Despite the absence of LGS, high concentrations of MPs induced dyslipidemia and NAFLD. Oral exposure to MPs triggered intestinal inflammation via natural killer cells, altered the gut microbiota, and modulated nutrient metabolism. Our study highlights the need for environmental measures to reduce oral MPs exposure in the future.

## 1 Introduction

Microplastics (MPs) are one of the most concerning pollutants worldwide. Plastics are one of the most widely produced materials, even amid the rigorous scrutiny of their environmental impacts (1). An estimated 8.3 billion tons of plastic has been produced to date (1). Despite requiring more energy than most other materials, plastic production, considering its current rate, is predicted to double within a few decades (2). Recently, MPs research has garnered considerable attention. MPs are small plastic particles less than 5 mm in diameter. Despite their small size, MPs pose a serious threat to various forms of life, including animals and plants, among other species. MPs are particularly prevalent in marine environments and cause significant harm to marine life (3). These minute particles are ingested by organisms such as fish, birds, and marine mammals and spread through the food chain within ecosystems. Additionally, MPs serve as carriers of environmental pollutants, accelerating the dissemination of harmful substances and potentially increasing the concentration of chemicals in water or soil, posing risks to ecosystems and human health.

MPs ingestion may lead to adverse health effects such as teratogenicity and mutagenicity anomalies (4, 5). MPs also exist in the soil, potentially affecting crops, wildlife, and vegetation. Moreover, the infiltration of these minute plastics into groundwater raises concerns regarding their impact on the drinking water supply and human health (6). The concentration of MPs (0.5–10 µm diameter) in plastic bottled mineral water was reported to be 656.8 µg/L ± 632.9 (7).

Plastics in the environment, owing to their downsizing, possess significant hydrophobicity, facilitating their pathways into the digestive systems of organisms, dissolution in lipid-containing digestive fluids, bioaccumulation, and incorporation into the food chain (8–10). Such indirect exposure and intrusion routes are believed to have the most profound impacts on humans. From a toxicological standpoint, the routes of exposure (oral, respiratory, or dermal) for the entry of MPs into the body are critical; oral exposure is the primary route. Pioneering studies on polystyrene MPs (PS-MPs) in mammals conducted by Deng et al. reported the accumulation of PS-MP particles in the liver, kidneys, and intestines in mice exposed to 5 or 20 μm fluorescent PS-MPs daily (11). Subsequent changes in metabolic profiles indicate that 5 μm PS-MPs affect energy metabolism, lipid metabolism, and oxidative stress in the mouse liver (11).

Furthermore, exposure of C57BL6/J mice to high concentrations of MPs increased the abundance and diversity of gut microbiota. Moreover, inflammation was reported in the intestines of mice exposed to high concentrations of MPs (12).

We previously reported that exposure to PS-MPs in C57BL6/J mice fed a regular diet (ND) or a high-fat diet (HFD) resulted in metabolic disorders, such as diabetes, dyslipidemia, and non-alcoholic fatty liver disease (NAFLD), in only HFD-fed mice, mediated by leaky gut syndrome (13). The innate immune response mediated by intestinal innate lymphoid cells (ILCs) affects the disruption of the mucosal barrier (14). Of the cells involved in the innate immune system, ILCs are a type of lymphocyte that form a part of the T cell-independent immune system. ILCs, including ILC1, ILC2, and ILC3, play crucial roles in regulating host responses to inflammation, tissue remodeling, tissue homeostasis, and infections (15). ILC3, which promotes mucin secretion from goblet cells through IL-22, is particularly important for maintaining mucosal barrier integrity in response to inflammation and infection (14).

Our previous study was limited because the lack of leaky gut syndrome at relatively low concentrations of PS-MPs (1000 μg/L) in ND-fed mice might have obscured a potential metabolic disorder. In this current study, we sought to explore the possibility of varying toxicity induced by PS-MPs at different concentrations. Therefore, to assess the toxicity of MPs in the absence of HFD-induced leaky gut syndrome, we investigated the effects of 1000 µg/L and a higher concentration, 5000 µg/L, of PS-MPs on alterations in the intestinal environment and metabolic disturbances in mice fed ND.

## 2 Materials and methods

### 2.1 Mice

**Figure 1:**
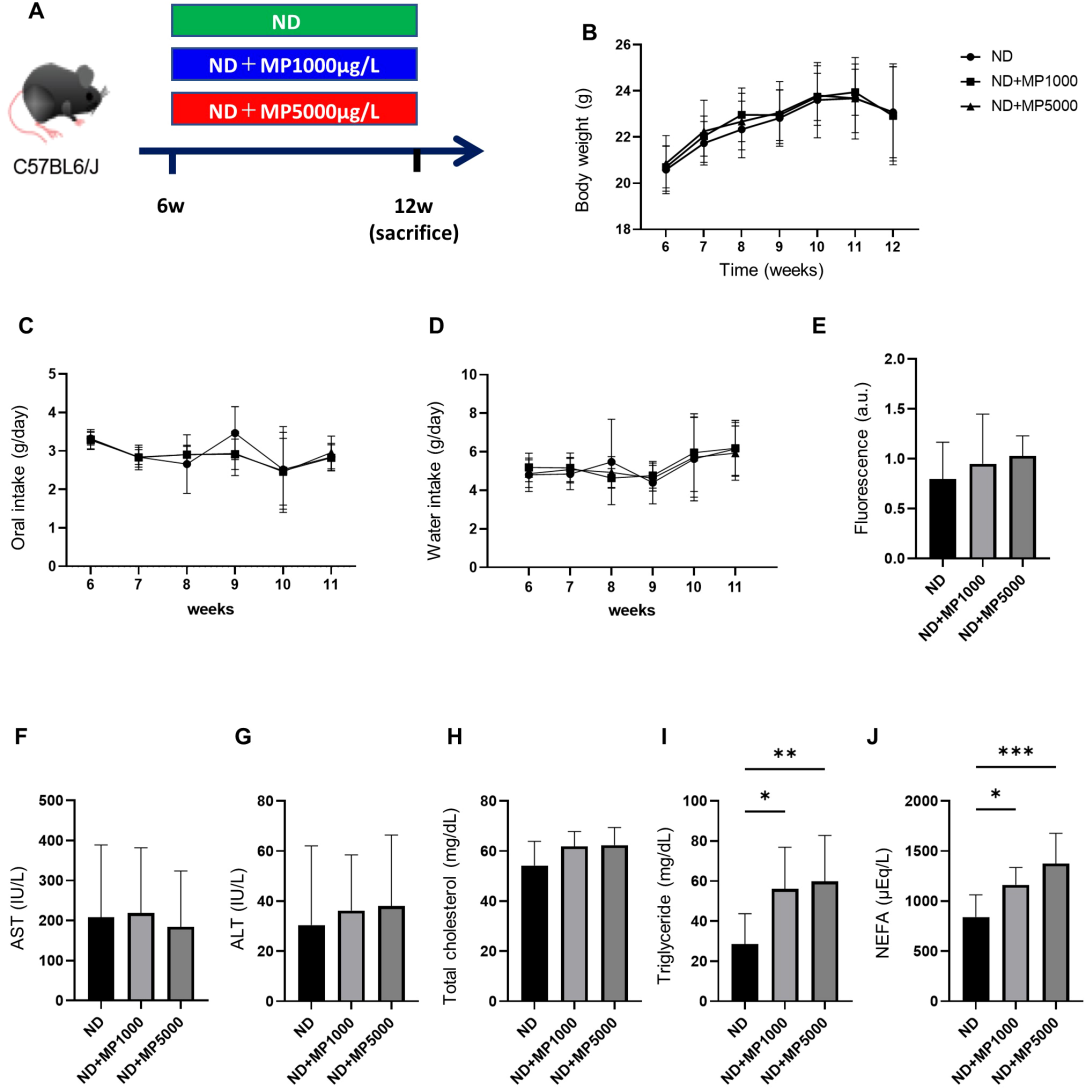
Effects of MP exposure on metabolic parameters and hepatic markers. **(A)** Exposure to ND ± MPs (1000 µg/L or 5000 µg/L) beginning at 6 weeks of age. **(B)** Changes in body weight (n = 10). **(C, D)** Changes in the intake of food and water (n = 10). **(E)** FITC-Dextran assay in 12-week-old mice (n = 10). Values are normalized to the levels of ND-fed mice. **(F–J)** Serum aspartate aminotransferase (AST), alanine aminotransferase (ALT), total cholesterol, triglyceride, and non-esterified fatty acid (NEFA) levels (n = 10). Data are presented as mean ± SD values. Data were analyzed using one-way ANOVA with Holm–Šídák’s multiple comparison test. *p < 0.05, **p < 0.01, and ***p < 0.001. MPs, microplastics; ND, normal diet; iPGTT, intraperitoneal glucose tolerance test; AUC, area under the curve; ITT, insulin tolerance test; FITC, fluorescein isothiocyanate.

All the experimental procedures were approved by the Committee for Animal Research, Kyoto Prefectural University of Medicine, Japan (approval number: M2023-87, M2022-92). Six-week-old C57BL/6J (wild-type) male mice were purchased from Shimizu Laboratory Supplies (Kyoto, Japan) and housed in a pathogen-free controlled environment. We used littermates born simultaneously in a mouse supply facility. The mice were housed one per cage and fed a normal diet (ND; 345 kcal/100 g, fat kcal 4.6%; CLEA, Tokyo, Japan) for a duration of six weeks, commencing at 6 weeks of age. Paired feeding was performed by supplying equal amounts of the feed. Mice in the MPs exposure group were administered carboxyl group-modified fluorescent polystyrene particles (F-K1 050; 0.45–0.53 μm polystyrene COOH; Green Fluorescent Protein (GFP) fluorescence; Merck, Germany) were dissolved in water at 1000 μg/L and 5000 μg/L under the condition of free water intake (n=10) (16, 17). Water with dissolved MPs was sonicated at 20 kHz for 15 minutes and the water was changed every 3 days, and the amount of water lost was measured to estimate the amount of water consumed (13). Body weight was assessed weekly. Upon reaching 12 weeks of age, the mice were euthanized by administering 0.3 mg/kg of medetomidine, 4.0 mg/kg of midazolam, and 5.0 mg/kg of butorphanol, after overnight fasting (18) and sacrificed under specific-pathogen-free conditions (**Figure 1A**).

### 2.2 Measurement of intestinal permeability

Five days before the mice were sacrificed, at the age of 12 weeks, mice were orally administered a solution of dextran labeled with fluorescein isothiocyanate (FITC; 25 mg/mL) at a dose of 20 mL/kg after a 4-hour fast. Blood samples were obtained through retro-orbital puncture, both prior to and 3 hours after oral gavage. The obtained blood samples were promptly subjected to density gradient centrifugation at 1500 × g for 15 minutes. The plasma fraction was harvested and diluted with phosphate-buffered saline (PBS) at a 1:2 ratio. Plasma levels of dextran were estimated by measuring luminescence using an Orion L microplate luminometer at an excitation wavelength of 490 nm and emission wavelength of 520 nm (Berthold Detection Systems, Pforzheim, Germany). A total of 10 samples were analyzed. Due to the challenges in distinguishing the FITC signal from FITC-dextran and the GFP signal from GFP-MPs through absorbance spectrometry, we assessed the GFP signal in the serum prior to administration and the FITC-dextran signal 3 hours after administration. The difference in plasma signal before and after FITC-dextran administration was calculated as a ratio to the control group and used as an indirect index of intestinal permeability (19).

### 2.3 Biochemical analysis

Blood samples were collected from fasted mice via the portal vein during sacrifice, and blood aspartate aminotransferase (AST), alanine aminotransferase (ALT), total cholesterol, triglyceride (TG), and non-esterified fatty acid (NEFA) levels were measured. Biochemical examinations were performed using the FUJIFILM Wako Pure 18 Chemical Corporation (Osaka, Japan).

### 2.4 Histological analysis of the jejunum, colon, and liver

Jejunum and colon were excised from mice, promptly immersed in 10% buffered formaldehyde, and allowed to fix for 24 hours at 22°C. Subsequently, they were embedded in paraffin, sectioned into 4-µm-thick slices, and stained with HE and periodic acid Schiff (PAS) stain in Carnoy’s solution.

Liver tissue was obtained, fixed in 10% buffered formaldehyde, embedded in paraffin, sectioned into 4-µm-thick slices, and subjected to staining with hematoxylin and eosin. Oil Red O stock solution was formulated in isopropanol (0.25 g/100 mL) and subjected to heating at 100°C for 10 minutes. The liver sections, fixed with 4% paraformaldehyde for 30 minutes and rinsed with PBS, were subsequently immersed in a 60% dilution of Oil Red O stock solution in distilled water for 30 minutes. Stained sections were further washed with PBS until the background was clear. Images of stained sections were captured using a fluorescence microscope (BZ-X810; Keyence). Villus height/width and crypt depth were quantified using HE-stained sections at five distinct sites per slide for each group of 10 animals using Image J (Version 1.53 k, NIH, Bethesda, MD, USA). Goblet cells (PAS+) were counted and expressed as the mean number of goblet cells (PAS+) per 10 crypts using Image J, following a previous study (20).

Additionally, to evaluate the severity of NAFLD, we computed the NAFLD activity score (NAS) (21), a widely recognized standard used for assessing non-alcoholic steatohepatitis (NASH) severity and measuring changes in NAFLD. The scoring system comprises 14 histological attributes, of which the following four were semi-quantitatively assessed: steatosis (0–3), lobular inflammation (0–2), hepatocellular ballooning (0–2), and fibrosis (0–4).

### 2.5 mRNA microarray analysis of the jejunum

The jejunum of mice subjected to a 16-hour fast was excised and promptly cryopreserved using liquid nitrogen. Both jejunum and liver specimens were homogenized in ice-cold QIAzol Lysis Reagent (Qiagen, Venlo, Netherlands), followed by isolation of total RNA according to the manufacturer’s instructions. A cDNA library was constructed using the TruSeq^®^ Stranded mRNA kit (Qiagen, Carlsbad, CA, USA). Paired-end sequencing was conducted on an Illumina NovaSeq6000 platform (n = 3). The comprehensive mRNA expression profiles of amino acids, fatty acids, and glucose transporters were visualized through volcano plots and heat maps.

### 2.6 Gene expression analysis in murine jejunum and liver

The jejunum and liver of mice fasted for 16 hours were excised and immediately frozen in liquid nitrogen. Total RNA was extracted for the microarray analysis.

Total RNA was reverse transcribed using a High-Capacity cDNA Reverse Transcription Kit (Applied Biosystems, Foster City, CA, USA) for first-strand cDNA synthesis using an oligonucleotide dT primer and random hexamer priming, according to the manufacturer’s recommendations. The reverse transcription reaction was carried out for 120 minutes at 37°C, followed by termination for 5 minutes at 85°C. Quantitative reverse transcription polymerase chain reaction (RT-qPCR) was used to quantify the mRNA expression levels of *Tnfa*, *Il6*, *Il1b*, *Il22*, *Pept1*, *Cd36*, and *Sglt1* in the jejunum and *Tnfa, I16*, *Ilib*, and *Mcp1* in the liver. RT-qPCR was performed using the TaqMan Fast Advanced Master Mix (Applied Biosystems, Waltham, MA, USA) according to the manufacturer’s instructions. The cycling conditions were as follows: 1 cycle for 2 min at 50°C and 20 s at 95°C, followed by 40 cycles of 1 s at 95°C and 20 s at 60°C.

The relative expression levels of each targeted gene were normalized to the threshold cycle (Ct) values of glyceraldehyde-3-phosphate dehydrogenase (GAPDH) and quantified using the comparative Ct 2^−ΔΔCT^ method. Signals originating from HFD-fed mice were assigned a relative value of 1.0. Six mice from each group were assessed, and RT-qPCR was conducted in triplicates for each sample.

### 2.7 Isolation of mononuclear cells from murine small intestine

To avert blood contamination in the jejunum, we conducted systemic perfusion using heparinized saline before the harvesting or rinsing of the tissue with PBS. The acquired samples were preserved in cold RPMI supplemented with 2% FBS until required. Upon euthanasia, mononuclear cells from the intestinal lamina propria (LPL) were isolated using the Lamina Propria Dissociation Kit (130-097-410; Miltenyi Biotec, Germany) following the manufacturer’s guidelines. The resultant cell pellets were subsequently suspended in 5 mL of 40% Percoll^®^ solution. This cellular suspension was delicately layered onto the upper segment of centrifuge tubes containing a lower layer of 5 mL of 80% Percoll^®^. Density gradient centrifugation was conducted at 420 × g for 20 minutes, and the mononuclear cells situated in the intermediary layer were meticulously extracted using a 1 mL pipette and then washed with 2% FBS/PBS (22).

### 2.8 Isolation of mononuclear cells from murine liver

Hepatic lymphocytes were isolated through mechanical dissection. Initially, the liver was excised from the euthanized mice, delicately filtered through a 200-gauge stainless-steel mesh, and suspended in Roswell Park Memorial Institute 1640 medium supplemented with 100 mL/L fetal calf serum (FCS, 10%). The suspension of liver cells was centrifuged at 1,500 rpm. The resulting pellet was then suspended in a 40% Percoll solution, layered onto an equivalent volume of 60% Percoll solution, and subjected to centrifugation at 2,000 rpm for 20 minutes at 22°C. Subsequently, cells were retrieved from the Percoll interface (buffy coat), pelleted through centrifugation, and rinsed twice with PBS containing 10% FCS prior to use (23).

### 2.9 Flow cytometry

The stained cells were analyzed using a FACSCanto II, and the resulting data were processed using FlowJo version 10.9.0 (TreeStar, Ashland, OR, USA). The following antibodies from eBioscience (San Diego, CA, USA) were used for gating of M1 and M2 macrophages: APC-CD45.2 (17045482; clone: 104, 1/50), PE-F4/80 (12480182; clone: BM8, 1/50), APC-Cy7-CD11b (47011282; clone: M1/70, 1/50), FITC-CD206 (MA516870; clone: MR5D3, 1/50), and PE-Cy7-CD11c (25011482; clone: N418, 1/50) (24) (**Supplementary Figure S1**).

The following antibodies from eBioscience were used for gating ILCs: biotin-CD3e (100304; clone: 145-2C11, 1/200), biotin-CD45R/B220 (103204; clone: RA3–6B2, 1/200), biotin-Gr-1 (108404; clone: RB6-8C5, 1/200), biotin-CD11c (117304; clone: N418, 1/200), biotin-CD11b (101204; clone: M1/70, 1/200), biotin-Ter119 (116204; clone: TER-119, 1/200), biotin-FceRIa (134304; clone: MAR-1, 1/200), FITC-Streptavidin (405202; 1/500), PE-Cy7-CD127 (135014; clone: A7R34, 1/100), Pacific Blue-CD45 (103116; clone: 30-F11, 1/100), PE-GATA-3 (clone: TWAJ, 1/50), APC-RORγ (clone: AFKJS-9, 1/50), and Fixable Viability Dye eFluor 780 (1/400) (25, 26) (**Supplementary Figure S2**).

### 2.10 Measurement of short-chain fatty acid levels in fecal samples

Short-chain fatty acid (SCFA) concentrations were assessed using gas chromatography–mass spectrometry (GC/MS) with an online solid-phase extraction (SPE) technique. In the SPE-GC system SGI-M100 (AiSTI Science, Wakayama, Japan), the sample was introduced into the vial and placed on the autosampler tray, and subsequently, SPE and injection into the GC/MS system were executed automatically. Solid phase stratification was accomplished using flash-SPE ACXs (AiSTI Science). For sample processing, a 50 µL aliquot of each extract was deposited onto the solid phase and then subjected to rinsing with a 1:1 mixture of acetonitrile and water. Following this step, the samples were dehydrated using acetone, impregnated with a 4 μL N-tert-butyldimethylsilyl-N-methyltrifluoroacetamide (MTBSTFA)-toluene solution (1:3 ratio) and eluted using hexane post-derivatization on the solid phase. The injection into the GC/MS system was carried out using a programmed temperature vaporizer (PTV) injector, LVI-S250 (AiSTI SCIENCE), with an initial temperature of 150°C for 0.5 minutes, gradually increasing to 290°C at a rate of 25°C/minute, and held for 16 min. The samples were injected into a capillary column, Vf-5ms (30 m × 0.25 mm (inner diameter) × 0.25 μm (membrane thickness); Agilent Technologies). The initial column temperature was 60°C for 3 minutes, increased by 10°C/minute to 100°C, further increased by 20°C/minute to 310°C, and finally held at 310°C for 7 minutes. Sample injection was performed in split mode at a ratio of 20:1. Each SCFA was identified using scan mode (m/z 70–470) (n=10) (27).

### 2.11 16S rRNA sequencing

Microbial DNA was isolated from frozen appendicular fecal samples using the QIAamp DNA Feces Mini Kit (Qiagen, Venlo, Netherlands), in accordance with the manufacturer’s protocols. Subsequently, the V3-V4 region of the 16S rRNA gene was amplified from the DNA using a bacterial universal primer set (341F and 806R). PCR was conducted using EF-Taq (Korea, Solgent), with 20 ng of genomic DNA serving as the template in a 30 µL reaction mixture. The thermocycling parameters were as follows: initial activation of Taq polymerase at 95°C for 2 minutes, followed by 35 cycles of denaturation at 95°C, annealing at 55°C, and elongation at 72°C for 1 minute each, and a final extension step at 72°C for 10 minutes. The PCR products were purified on a multiscreen filter plate (Millipore Corp., Billerica, MA, USA). A MiSeq sequencer (Illumina, CA, USA) was used for 16S rRNA sequencing according to the manufacturer’s instructions (Macrogen, Seoul, Korea). For quality filtering of the sequences, QIIME version 1.9.1 (28) was used. Barcodes or primers with scores of less than 75% were excluded from the files. Operational taxonomic units (OTUs) with 97% sequence similarity were determined using the UCLUST algorithm (29). Moreover, BLAST (UNITE database v9.0, released in 2023. Available online: https://unite.ut.ee) was applied for the taxonomic classification of 16S rRNAs, using the UNITE sequence set of the Greengenes core set aligned with UCLUST and ITS.

Kyoto Encyclopedia of Genes and Genomes (KEGG) ortholog abundances were predicted using Phylogenetic Investigation of Communities by Reconstruction of Unobserved States (PICRUSt2) software (30).

The relative abundance of phyla within different groups was assessed using one-way ANOVA with a Holm–Šídák multiple-comparison test. Alpha diversity, representing diversity within individual samples, was evaluated using the Chao1 (31), Shannon (32), and Gini–Simpson indices (33).

The relative abundance of bacterial genera between groups was assessed using linear discriminant analysis (LDA) coupled with effect size measurements (LEfSe) (http://galaxy.biobakery.org/, accessed November 16, 2023) (34). Using a normalized relative abundance matrix, taxa exhibiting statistically significant differences in abundance were discerned using LEfSe, with the effect size of these features being determined via LDA. The significance level was set at *p* ≤ 0.05 using the Wilcoxon rank-sum test, and an effect size threshold of 2 was applied to all biomarkers.

### 2.12 Statistical analysis

Data were analyzed using JMP software (version 14.0; SAS, Cary, NC, USA). One-way ANOVA with Holm–Šídák’s multiple comparisons test was used to compare the results of different groups. Data are presented as mean ± SD values. Statistical significance was set at P < 0.05. Figures were created using the GraphPad Prism software (version 10.1.2; San Diego, CA, USA).

## 3 Results

### 3.1 Effects of MPs exposure on body weight and intestinal permeability

From 8 to 14 weeks of age, body weight, and food and water intake were monitored in ND, ND + MP 1000 µg/L, and ND + MP 5000 µg/L mice. There were no discernible disparities in body weight between mice in these groups (**Figure 1B**).

**Figure 2:**
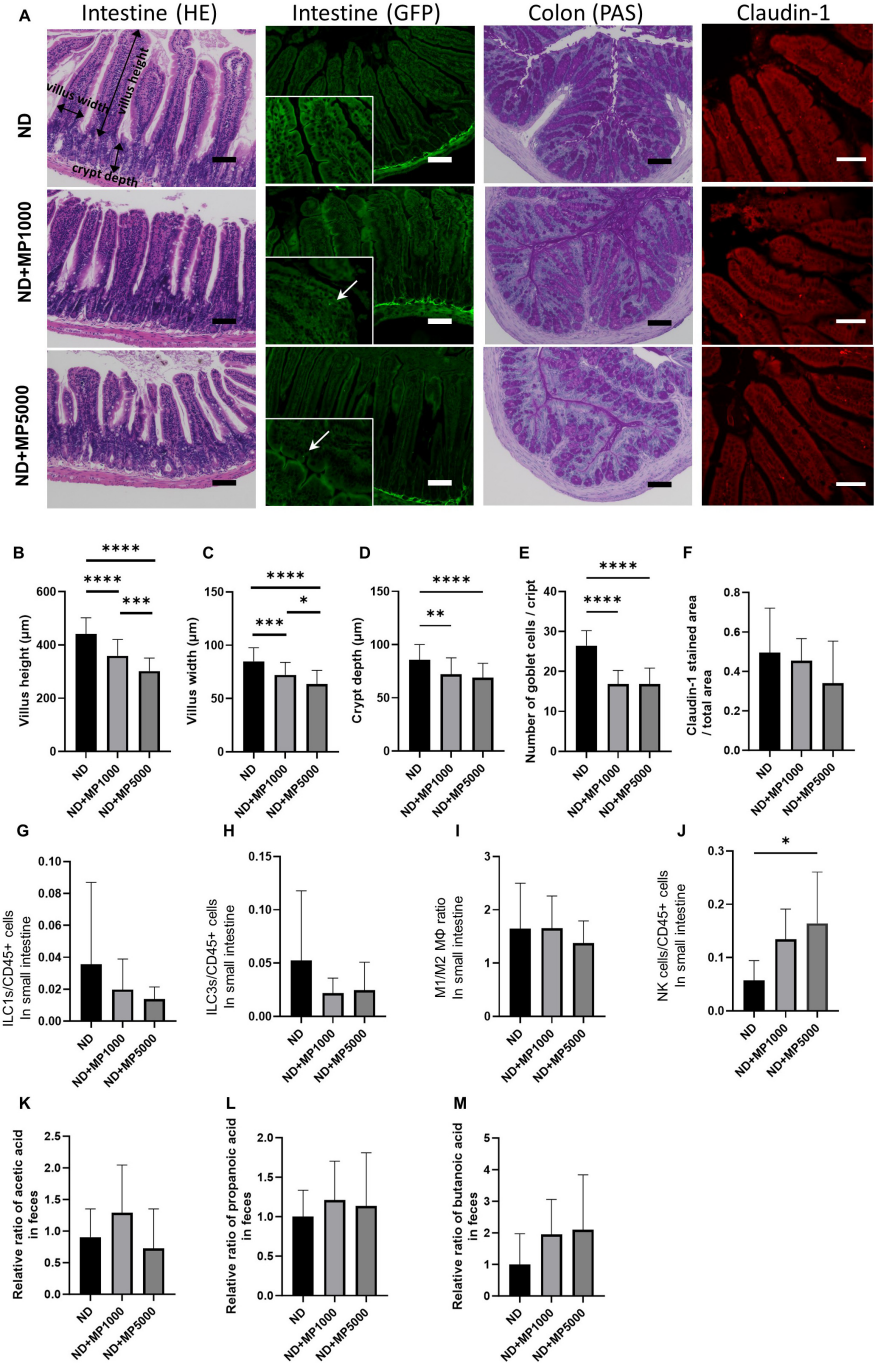
Effects of MP exposure on histological and molecular parameters of jejunum. and colon tissues. **(A)** Representative images of hematoxylin & eosin (HE)-stained and green fluorescent protein (GFP)-labeled jejunum sections, periodic acid Schiff (PAS)-stained colon sections and Claudin1-immunostained jejunum sections. In the GFP fluorescence image, MPs are enlarged and indicated by arrows. Scale bars indicate 100 μm (50 µm for Claudin1 image). **(B)** Villus height (n = 10). **(C)** Villus width (n = 10). **(D)** Crypt depth (n = 10). **(E)** Total goblet cells/crypt (n=10). **(F)** Claudin1 stained area/total area (n = 10). Percentages of **(G)** ILC1s to CD45-positive cells, **(H)** ILC3s to CD45-positive cells, **(I)** M1 macrophages to M2 macrophages, and **(J)** NK cells in the small intestine (n = 10 in each case). Relative ratios of **(K)** concentration of acetic acid, **(L)** propanoic acid, and **(M)** butanoic acid relative to the mean concentrations in ND mice. Data are presented as mean ± SD values. Data were analyzed using one-way ANOVA with Holm–Šídák’s multiple comparison test. *p < 0.05, **p < 0.01, ***p < 0.001, and ****p < 0.0001. MPs, microplastics; ILCs, innate lymphoid cells; NK, natural killer.

There were no differences in food or water intake between the three groups (**Figures 1C, D**). Intestinal permeability was evaluated using fluorescent-labeled dextran. There existed no significant difference in plasma FITC expression between ND mice, ND + MP 1000 µg/L mice, and ND + MP 5000 µg/L mice (**Figure 1E**). We also assessed the immunostained area of the small intestine for claudin-1, a crucial membrane protein constituting tight junctions. While there was a tendency for the Claudin-1 staining area to decrease, no statistically significant difference was discerned among ND mice, ND + MP 1000 μg/L mice, and ND + MP 5000 μg/L mice (**Figures 2A, F**).

### 3.2 Effects of MPs exposure on serum levels of liver enzymes and lipids

We investigated serum levels of hepatic enzymes and lipids. There were no significant differences in serum AST, ALT, and total cholesterol levels between ND mice, ND + MP 1000 µg/L mice, and ND + MP 5000 µg/L mice, but TG and NEFA were significantly higher in ND + MP 1000 µg/L and ND + MP 5000 µg/L mice than in ND mice (**Figures 1F–J**).

### 3.3 Effects of MPs exposure on jejunal histology and innate immunity

Representative histological images of the jejunum are shown in **Figure 2A**. The villus height and width were significantly lower in ND + MP 1000 μg/L and ND + MP 5000 μg/L mice than in ND mice (**Figures 2B, C**); therefore, the decrease in villus height and width due to MPs tended to be smaller with increasing concentrations. Furthermore, crypt depth was reduced in ND + MP1000 µg/L and ND + MP 5000 µg/L mice than in ND mice (**Figure 2D**). The total number of goblet cells was determined using the PAS-stained images. The number of goblet cells was significantly lower in ND + MP 1000 µg/L and 5000 µg/L mice than in ND mice (**Figure 2E**).

We also measured the number of cells involved in innate immunity in the intestinal LPL using FACS. Although there were no significant changes in ILC1, ILC3, and M1/M2 macrophage ratios in the intestinal LPL due to MPs exposure (**Figures 2G–I**), the number of NK cells was higher in ND + MP 5000 µg/L mice than in ND mice (**Figure 2J**).

The concentration of SCFA in the feces was measured using GC/MS and was expressed as the ratio to the mean concentration in the feces of ND mice. SCFA such as acetic acid (**Figure 2K**), propanoic acid (**Figure 2L**), and butanoic acid (**Figure 2M**) were not significantly different between any of the groups.

### 3.4 Effects of MPs exposure on the intestinal expression of genes related to inflammation and transporters

**Figure 3:**
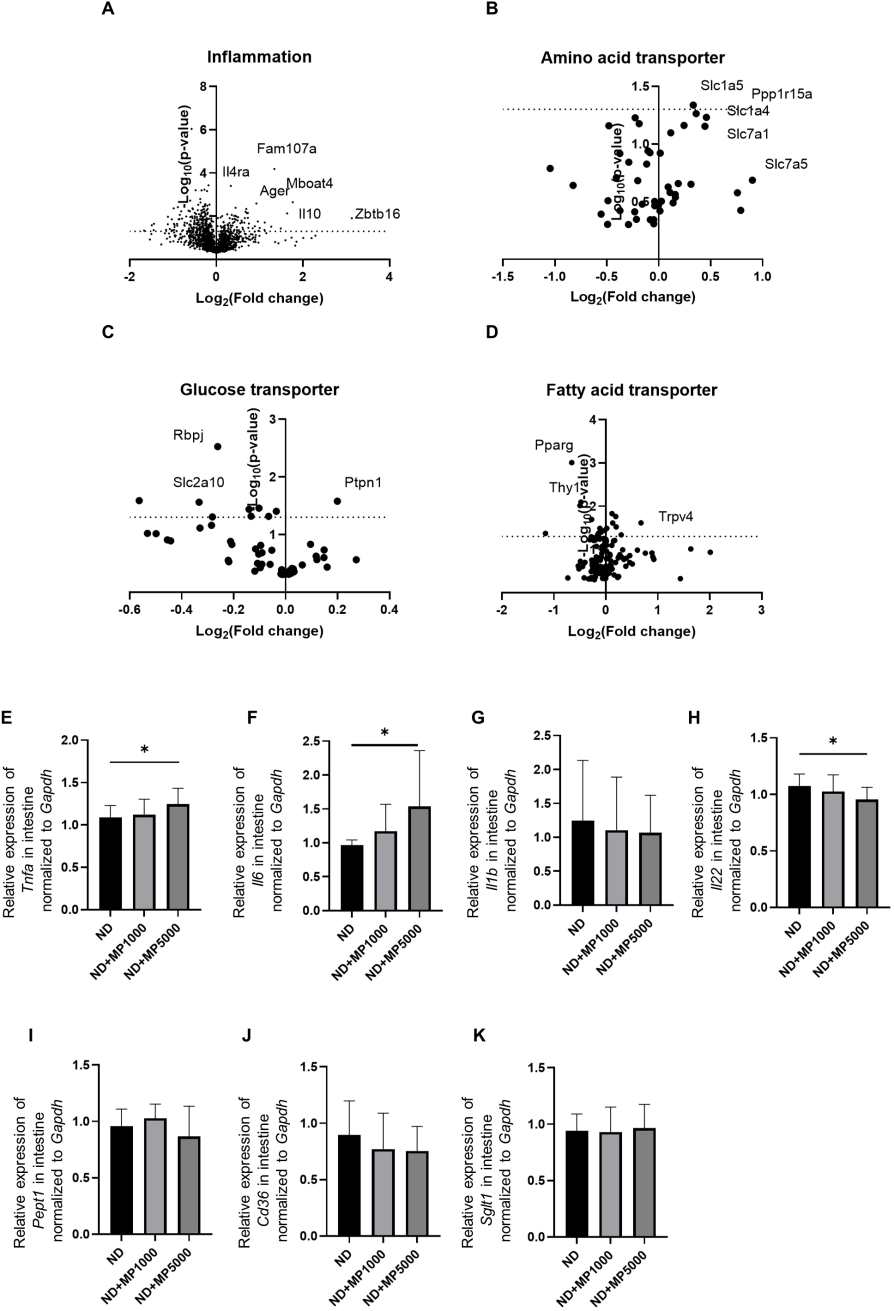
Changes in the expression of genes involved in intestinal inflammation and nutrient absorption. Global mRNA expression of gene related to **(A)** inflammation, **(B)** amino acid transporters, **(C)** glucose transporters, and **(D)** fatty acid transporters visualized as a volcano plot (n=3). Relative mRNA expression of **(E)** *Tnfa*, **(F)** *Il6*, **(G)** *Il1b*, **(H)** *Il22*, **(I)** *Pept1*, **(J)** *Cd36*, and **(K)** *Sglt1* in the intestine normalized to the expression of *Gapdh* (n = 10). Data are presented as mean ± SD values. Data were analyzed using one-way ANOVA with Holm–Šídák’s multiple comparison test. *p < 0.05.

Differences in the expression of several transporters and metabolism-related genes in the small intestine were determined using microarray analysis. The expression of inflammation-related genes such as *FAM107a*, *Il4ra*, *Ager*, *Mboat4*, *Il10*, and *Zbtb16* was higher in ND + MP 5000 µg/L mice than in ND mice (**Figure 3A**).

The expression of glucose transporter genes, such as *Ptpn1* was higher, whereas that of *Rbpj* and *Slc2a10* was lower (**Figure 3B**) in ND + MP 5000 µg/L mice than in ND mice. The expression of amino acid transporter genes, such as *Ppp1r15a*, *Slc1a4*, and *Slc7a1*, was higher, whereas that of *Rbpj* and *Slc2a10* was lower in ND + MP 5000 µg/L mice than in ND mice (**Figure 3C**). The expression of fatty acid transporter gene *Trpv4* was higher whereas that of *Pparg* and *Thy1* was lower in ND + MP 5000 µg/L mice than in ND mice (**Figure 3D**).

Gene expression in the small intestines of each group was compared using real-time PCR. The expression of genes associated with inflammation, such as *Tnfa* and *Il6*, was higher (**Figures 3E, F**), while that of *Il22* related to intestinal immunity (35) was significantly lower (**Figure 3H**) in ND + MP 5000 µg/L mice than in ND mice. No significant differences were observed in the expression of *Il1b* associated with inflammation and *Pept1*, *Cd36*, and *Sglt1* related to nutrient absorption, in the small intestine among any of the groups (**Figures 3G, I-K**).

### 3.5 Effects of MPs exposure on liver histology and immune cell composition

**Figure 4:**
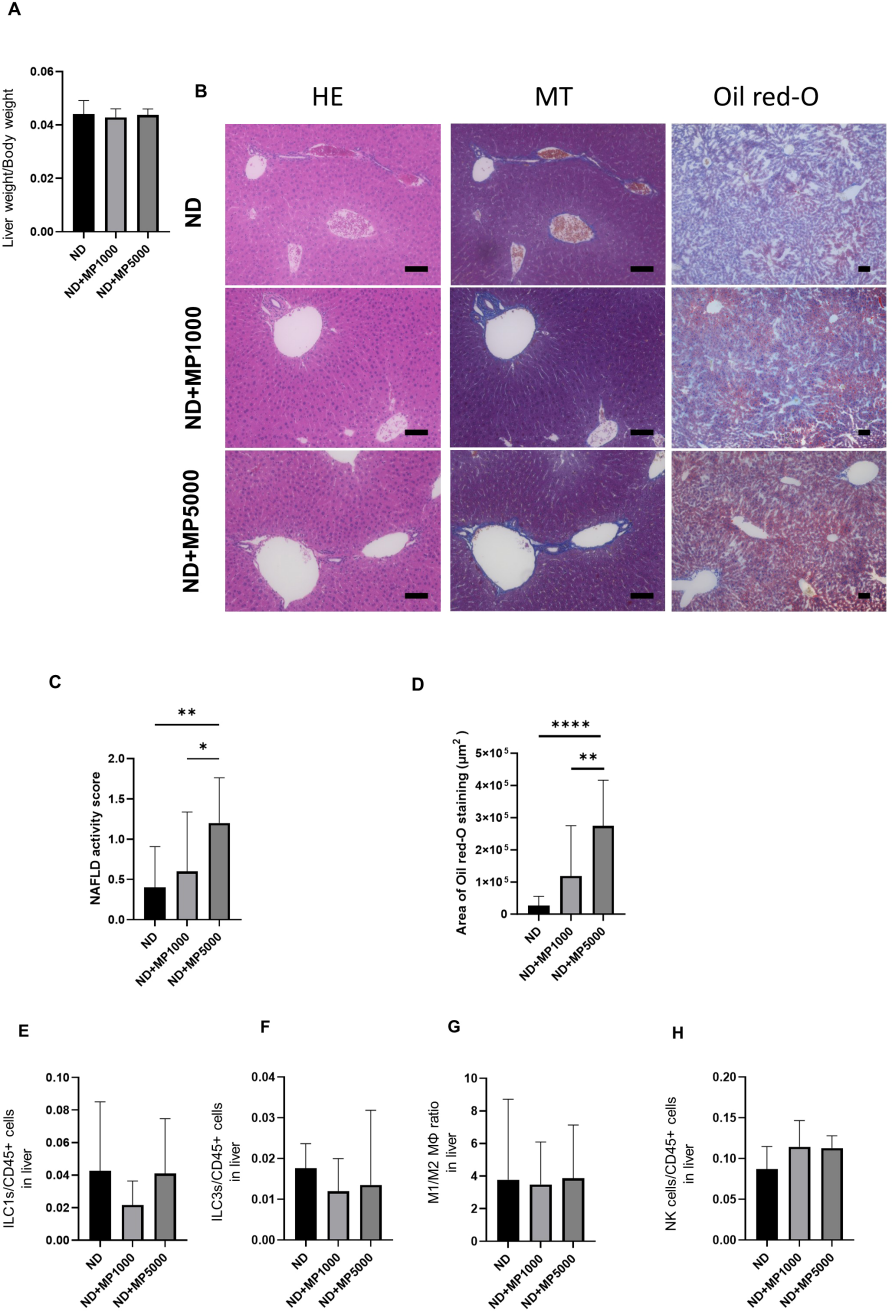
Histological evaluation of liver and immune cells involved in innate immunity. **(A)** Relative liver weight (n=10). **(B)** Representative images of hematoxylin & eosin (HE)- and Oil red-O-stained liver sections. Scale bars indicate 100 μm. **(C)** Non-alcoholic fatty liver disease (NAFLD) activity scores (n = 10). **(D)** Area of Oil red-O-stained region (n = 10). Percentages of **(E)** ILC1s to CD45-positive cells, **(F)** ILC3s to CD45-positive cells, **(G)** M1 macrophages to M2 macrophages, and **(K)** NK cells in the small intestine (n = 10 in each case). Data are presented as mean ± SD values. Data were analyzed using one-way ANOVA with Holm–Šídák’s multiple-comparisons test. *p < 0.05, **p < 0.01 and ****p < 0.0001. MPs, microplastics; ILCs, innate lymphoid cells; NK, natural killer.

Liver weight / body weight was not significantly different between any of the groups (**Figure 4A**). Liver histology was analyzed to investigate the effects of MPs on the liver. Representative histological images of the liver are shown in **Figure 4B**. The NAFLD activity score and Oil Red staining area were higher in ND + MP 5000 μg/L mice than in ND mice and ND + MP 1000 μg/L mice. However, there were no significant differences between ND + MP 1000 μg/L mice and ND mice (**Figures 4C, D**).

The number of cells involved in innate immunity in the liver was measured by FACS. There were no significant differences in the number of ILC1 and ILC3, the ratio of M1/M2 macrophages, or NK cells due to MPs exposure between any of the groups (**Figures 4E–H**).

### 3.6 Effects of MPs exposure on the hepatic expression of genes related to inflammation and hepatic steatosis

**Figure 5:**
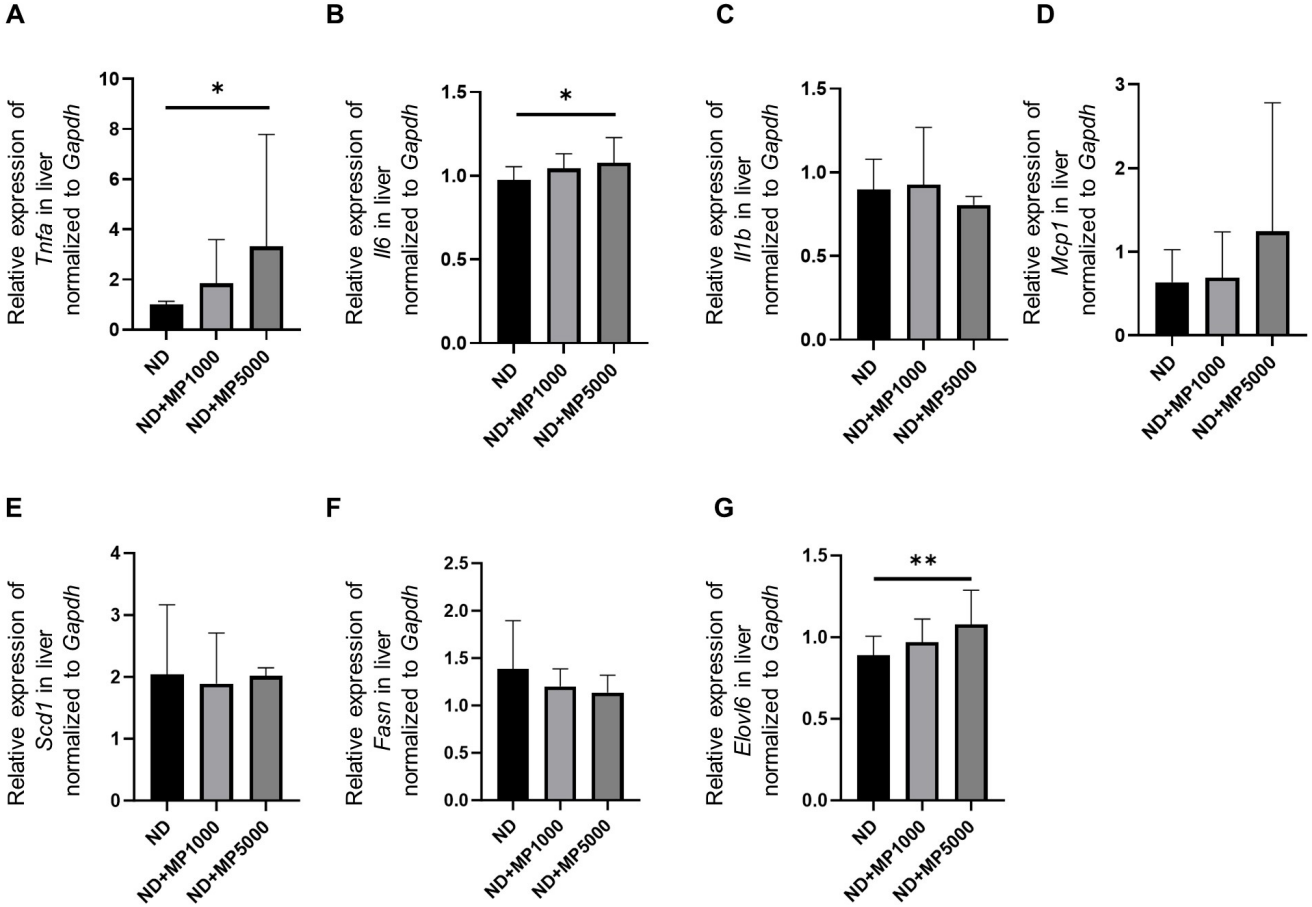
Changes in the expression of genes involved in liver inflammation and hepatic steatosis. The relative mRNA expression of **(A)** *Tnfa*, **(B)** *Il6*, **(C)** *Il1b*, **(D)** *Mcp1*, **(E)** *Scd1*, **(F)** *Fasn*, and **(G)** *Elovl6* in the liver normalized to the expression of *Gapdh* (n = 10). Data are presented as mean ± SD values. Data were analyzed using one-way ANOVA with Holm–Šídák’s multiple comparison test. *p < 0.05 and **p < 0.01.

The expression of genes related to inflammation and hepatic steatosis in the liver of each group was compared using real-time PCR. Compared to ND mice, the expression of inflammation-related genes *Tnfa* and *Il6* was not different in ND + MP 1000 µg/L mice but significantly higher in ND + MP 5000 µg/L mice (**Figures 5A, B**). In contrast, no differences were observed in the expression of inflammation-related genes *Il1b* and *Mcp1* between any of the groups (**Figures 5C, D**). Further, the expression of hepatic steatosis-related genes *Scd1* and *Fasn* in any of the groups, while that of *Elovl6* was higher in ND + MP 5000 µg/L mice than in ND mice (**Figures 5E–G**).

### 3.7 Effects of MPs exposure on gut microbiota composition

**Figure 6:**
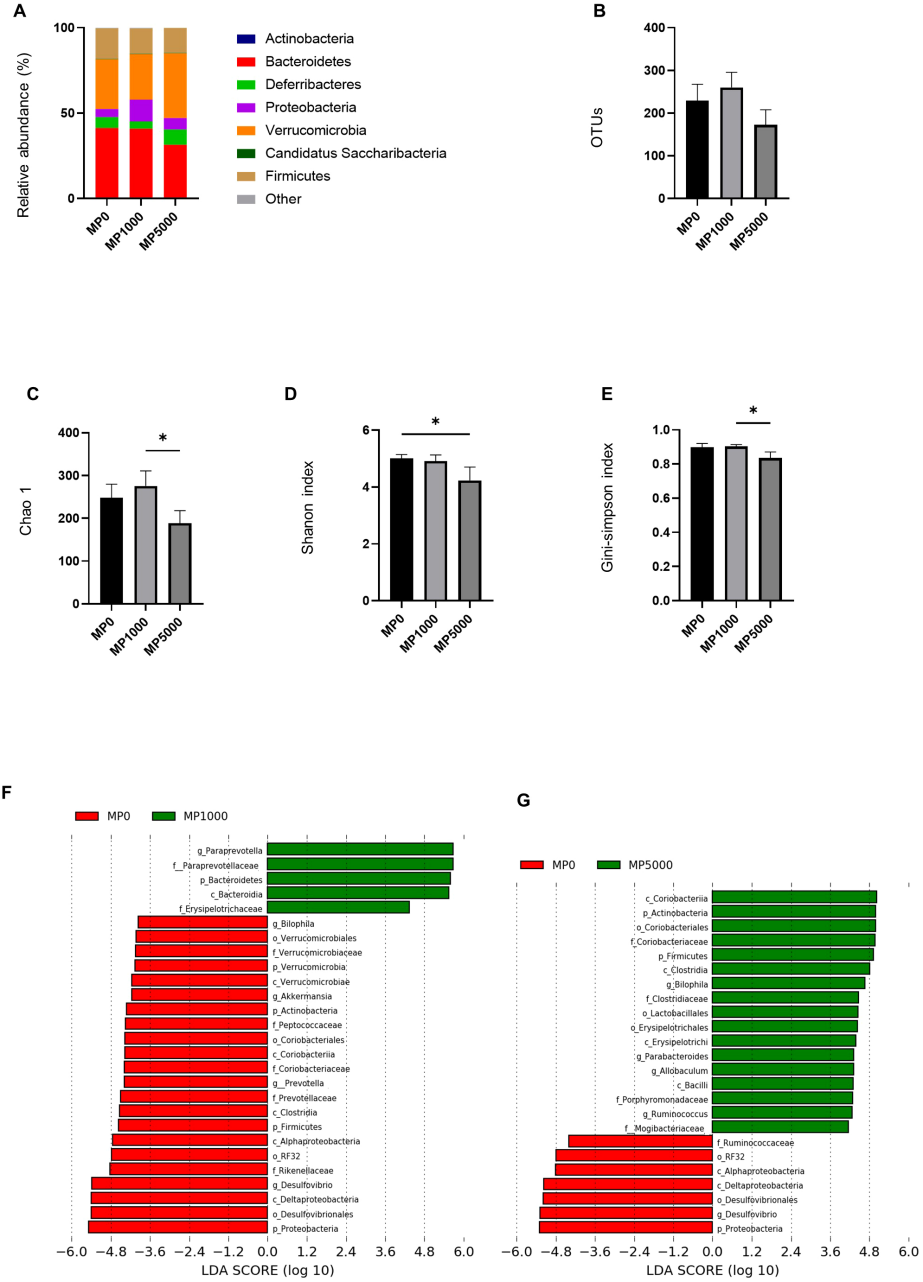
16S rRNA sequencing of gut microbiota of mice exposed to MPs. **(A)** Relative abundance of phyla (%). **(B)** The number of operational taxonomic units (OTUs). **(C)** Chao1 index. **(D)** Shannon index. **(E)** Gini-Simpson index. **(F)** Linear discriminant analysis (LDA) score (Log10) of ND and ND+MP 1000 µg/L mice; (Red) taxa enriched in ND mice; (Green) taxa enriched in ND+MP 1000 µg/L mice. **(G)** LDA score of ND and ND+MP 5000 µg/L mice; (Red) taxa enriched in ND mice; (Green) taxa enriched in ND+MP 5000 µg/L mice. Only taxa with a significant LDA threshold value >2 are shown. Data are presented as mean ± SD values. Data were analyzed using one-way ANOVA with Holm–Šídák’s multiple comparison test. *p < 0.05. ND, normal diet; LDA, LDA coupled with effect size measurements; MPs, microplastics.

We investigated the relative abundance of gut microbiota using 16s rRNA sequencing (**Figure 6A**). There was a decrease in the relative abundance of phylum Bacteroides between ND mice and ND + MP 5000 µg/L mice. There were no differences in OTUs between ND mice, ND + MP 1000 µg/L mice, and ND + MP 5000 µg/L mice (**Figure 6B**). However, in terms of alpha diversity indices, the Shanon index was significantly lower, and the Chao1 index and Gini-Simpson index showed a decreasing trend in ND + MP 5000 µg/L mice than in ND mice. No differences were observed in the alpha diversity indices between ND mice and ND + MP 1000 µg/L mice (**Figures 6C-E**).

Furthermore, taxa with considerably different abundances between ND mice and ND + MP 1000 µg/L mice, and ND mice and ND + MP 5000 µg/L mice were identified using the LEfSe algorithm. Five taxa (including the genus *Paraprevotella*, the family *Paraprevotellaceae*, and the family *Erysipelotrichaceae*) were significantly over-represented, while 22 taxa (including the genus *Akkermansia*, the family *Verrucomicrobiaceae*, the genus *Prevotella*, the family *Prevotellaceae*, the family *Rikenellaceae*, the order *RF32*, and the genus *Desulfovibrio*) were under-represented in ND + MP 1000 µg/L mice than in ND mice (**Figure 6F**). Taxa (including the family *Coriobacteriaceae*, the genus *Biophilia*, the genus *Allobaculum*, and the phylum *Firmicutes*) were over-represented, while seven taxa (including the order *RF32* and the genus *Desulfovibrio*) were under-represented in ND + MP 5000 µg/L mice than in ND mice (**Figure 6G**).

## 4 Discussion

In this study, we investigated the effect of MPs exposure in mice fed a ND. Exposure to high concentrations of MPs increased serum lipid concentrations and exacerbated fatty liver disease, even in the absence of leaky gut syndrome caused by a high-fat diet. Intestinal tracts exposed to MPs did not show changes in spontaneous lymphocyte counts or SCFA, but caused increases in NK cells, changes in the intestinal flora, inflammation, and gene expression related to nutrient transport in the gut; the degree of impairment of the intestinal environment was MPs dose-dependent.

Plastic particles cause multifaceted problems across various environmental domains. Humans are exposed directly to MPs in drinking water, sea salt, and the atmosphere or indirectly through the food chain (36–38). Bottled water, in particular, contains a significant number of MPs (39, 40), and its continuous intake through regular drinking raises concerns. The findings of this study contribute to the evaluation of the potential toxicity of MPs, especially at high concentrations.

A previous study has reported that MPs intake per adult ranges from 0.1–5 g/day (37), which corresponds to approximately 0.2–10.2 mg/kg BW/day for a 70 kg adult. In our current mouse model, MPs exposure was via drinking water only, corresponding to calculated doses of 0.2 mg/kg BW/day and 1.0 mg/kg BW/day. Stock et al. (41) previously used a dosing regimen of PS-MPs at < 34 mg/kg BW, administered three times a week for 4 weeks. They reported minimal particle uptake in intestinal tissues without toxic effects (41). In our previous study (13), no metabolic abnormalities due to MPs exposure were observed in ND-fed mice compared with HFD-fed mice. However, toxicity due to MPs exposure was observed in the present study. This may be attributed to the exacerbation of inflammation and alteration in the gut microbiota because of the continuous and higher concentration exposure to MPs, resulting in its accumulation in the intestines.

In this study, we observed an increase in serum triglycerides and NEFA due to MPs exposure, consistent with the findings of Deng et al. (11), who highlighted the impact of daily administration of 5 µm PS-MPs on liver energy metabolism, lipid metabolism, and oxidative stress in mice. Furthermore, we analyzed the dynamics of immune cells involved in the innate immunity of the small intestine. Previous studies have reported changes in the number of ILC3 cells in case of a disrupted mucosal barrier in mice with colitis (42). Additionally, an increase in the number of inflammatory cells, such as ILC1s and M1 macrophages, in the lamina propria of the small intestine, along with a decrease in SCFA production in feces (35), has been reported in *db/db* mice. However, in our study, although the numbers of ILCs and macrophages in the intestines of mice exposed to MPs remained unchanged, there was an increase in NK cells in the group exposed to high concentrations of MPs (5000 µg/L). NK cells secrete IFN-γ and TNF-α, triggering inflammation (43). Although changes in NK cells in the intestine due to MPs exposure have not been previously reported, existing studies have suggested that MP-induced inflammation in the intestine may involve NK cells (12, 44), indicating their potential involvement in the inflammatory response triggered by MPs in the intestine.

In the present study, no significant differences were found in SCFA levels after MPs exposure in mice fed a ND, which is consistent with our previous report (13). The production of SCFA from intestinal bacteria decreases during inflammation (45), but SCFA production did not decrease during this 6-week exposure period. This result was supported by the lack of a difference in the number of ILC3s that use SCFA as substrate. Propanoic acid and butanoic acid have been reported to maintain homeostasis of the intestinal microbiota in the intestinal tract (46); their production likely did not exhibit a compensatory decrease in this study. However, the specific mechanism behind this phenomenon requires further investigation.

Expression of inflammation-related genes was altered in the small intestine exposed to high concentrations of MPs. Upregulation of *Ager* which encodes the receptor for advanced glycation end-products (RAGE), leads to an unfavorable pro-inflammatory state associated with inflammatory diseases, autoimmune disorders, infections, diabetes, metabolic syndrome and its complications, obesity, insulin resistance, hypertension, atherosclerosis, neurological disorders such as Alzheimer’s disease, cardiovascular diseases, and various other conditions through multiple pathways (47–49). Additionally, *Mboat4* encodes ghrelin-O-acyltransferase (GOAT), which has been reported to be overexpressed in colitis (50). Furthermore, the upregulation of *Ptpn1* encoding PTP1B may have influenced liver steatosis. Since PTP1B-deficient mice are protected from diet-induced obesity and hepatic steatosis, the upregulation of Ptpn1 might, conversely, not protect against hepatic steatosis and could potentially contribute to its development (51, 52). *Rbpj* encodes the Notch-1 pathway transcription factor RBP-J (recombinant binding protein suppressor of hairless), and dysfunction of RBP-J has been associated with the loss of epithelial barrier integrity and abnormal conversion of proliferative crypt cells into goblet cells (53). *Slc2a10* encodes GLUT10, which regulates connective tissue formation and adipogenesis through ascorbic acid-dependent DNA demethylation. Its deficiency can lead to abnormal connective tissue formation and a reduced protective effect against HFD-induced metabolic dysregulation (54, 55).

Upregulation of *TRPV4* has been associated with inflammation in patients with ulcerative colitis and the colitis mouse model (56, 57), and the TRPV4 channel may potentially increase vascular permeability in colitis inflammation. TRPV4 is speculated to be involved in IL-6 and IL-8 production via ATP release at the onset of inflammation (58). Moreover, a decrease in the expression of *Pparg*, as reported by Lu et al. (56), was also observed in this study. They reported increased PPARα expression and decreased PPARγ expression at the mRNA level, accompanying the downregulation of hepatic triglyceride synthesis in mice exposed to 0.5 and 50 μm PS-MPs (100 and 1000 μg/L) through drinking water for 35 days. Despite observing changes in genes related to inflammation and metabolism, no significant alterations were detected in *Pept1, Cd36, and Sglt1* using RT-PCR. However, in this study, we focused on nutrient absorption through the expression of genes involved in nutrient metabolism and transporters.

Mice exposed to MPs showed exacerbated fatty liver, whereas the number of ILCs and macrophages remained unchanged. However, an increase in liver NK cells was observed in the group exposed to a high MPs concentration (5000 µg/L). Zhao et al. (57) reported increased infiltration of NK cells into the liver tissues, decreased expression of PD-1 of NK cells, and an increase in M1 macrophages, promoting inflammation in mice administered 0.5µm PS-MPs at a dose of 0.5 mg/day. Our results are in line with the decrease in NK cells but not the increase in macrophages; the increase in macrophages observed by Zhao et al. could be attributed to the higher dosage of MPs used in their study than in our experiment (57). The ND+MP 5000 µg/dL mice showed increased expression of *Elovl6*, which encodes an enzyme involved in the elongation of fatty acids, implicated in NASH (59). Increased expression of Elovl6 may contribute to the promotion of hepatic steatosis.

In addition, alterations in gut microbiota were observed in the intestines of mice exposed to MPs. Mice exposed to high concentrations of MPs exhibited a reduced relative abundance of the phylum Bacteroides along with a decrease in alpha diversity, indicating dysbiosis. Previous studies have also reported reduced diversity of intestinal bacteria in MP-exposed mice (56, 60), which aligns with our results. Compared to ND mice, the abundances of the family *Paraprevotellaceae*, which is related to IL-6 (61), and the family *Erysipelotrichaceae*, associated with NASH and dysbiosis (62), were significantly increased in ND+MP 1000 µg/dL mice. In contrast, compared to ND mice the abundances of the family *Verrucomicrobiaceae*, which is negatively correlated with inflammation and obesity (63), and the order *RF32*, which is positively correlated with the improvement of lipid abnormalities (64), was decreased in ND+MP 1000 µg/dL mice. Compared to ND mice, the abundances of the genus *Allobaculum*, which is related to NASH and dysbiosis (62), and the phylum *Firmicutes*, which is associated with inflammation (65), were significantly increased, whereas the abundance of the order *RF32*, which typically increases with the improvement of lipid abnormalities (64), was decreased in ND+MP 5000 µg/dL mice.

In conclusion, exposure to MPs induced a dose-dependent intestinal inflammation mediated by NK cells, alterations in the gut microbiota, and disruption of the gut environment. Consequently, changes in the intestinal environment altered the expression of genes associated with nutrient metabolism. Therefore, even in the absence of HFD-induced leaky gut syndrome, exposure to high concentrations of MPs disrupted lipid metabolism and aggravated fatty liver. This study emphasizes the need for environmental measures to reduce oral exposure to MPs and highlights the need for further clinical research on MPs implications.

## Data Availability

The datasets presented in this study can be found in online repositories. The names of the repository/repositories and accession number(s) can be found below: PRJNA1110833 (SRA).
