# Supplementary material for: Oral exposure to high concentrations of polystyrene microplastics alters the intestinal environment and metabolic outcomes in mice
Source: Front Immunol. 2024 Nov 12;15:1407936. doi: 10.3389/fimmu.2024.1407936 (PMC11588728; doi:10.3389/fimmu.2024.1407936)
Supplement: Supplementary file 1 [file DataSheet1.docx]

Supplementary Material

# Supplementary Data

None.

# Supplementary Figures and Tables

For more information on Supplementary Material and for details on the different file types accepted, please see [here](https://www.frontiersin.org/guidelines/author-guidelines#supplementary-material).

## Supplementary Figures


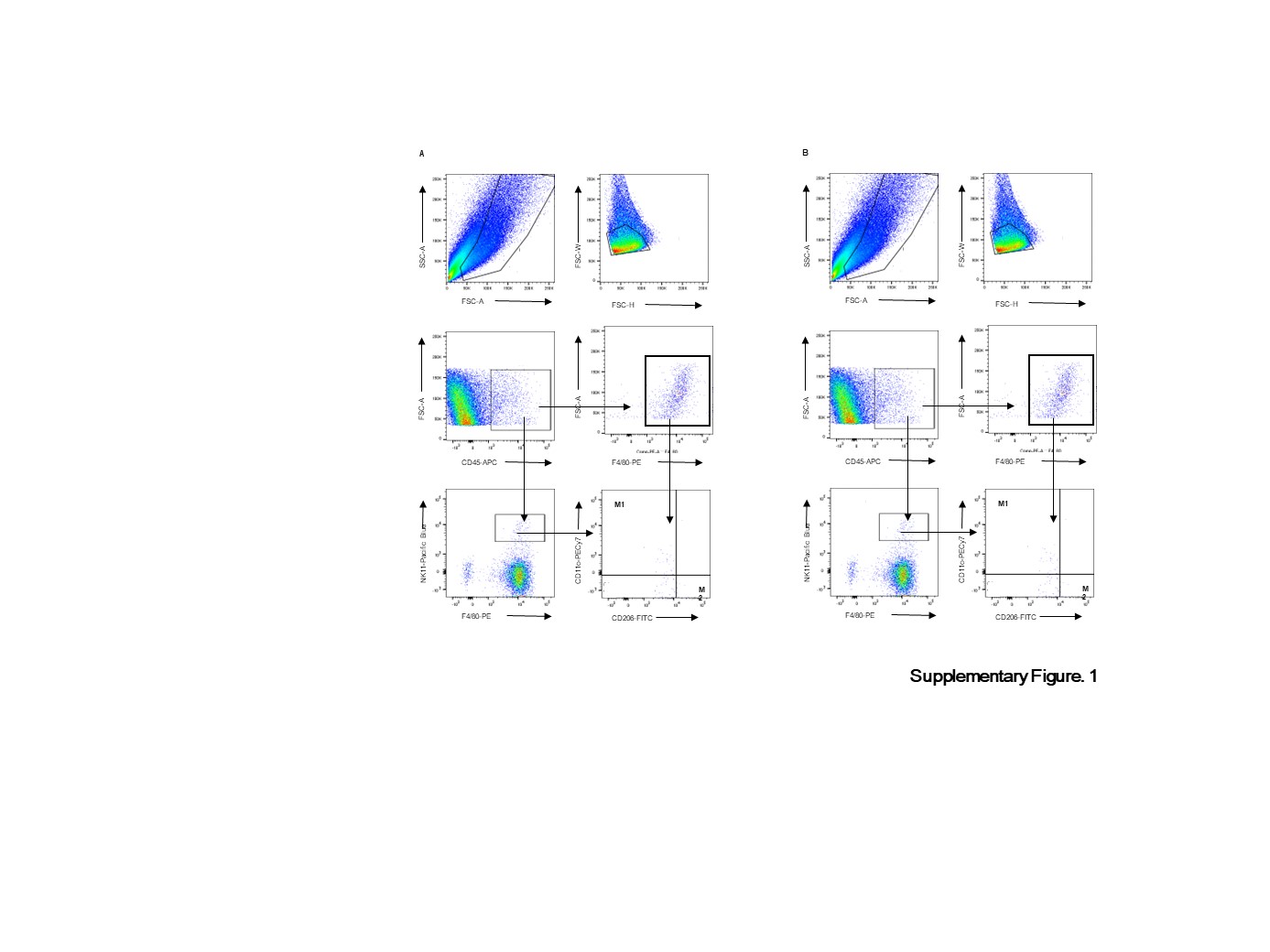


**Supplementary Figure 1.** **Gating strategy of macrophages.**

Representative flow cytometry plots of macrophages in the intestine (1A) and the liver (1B).


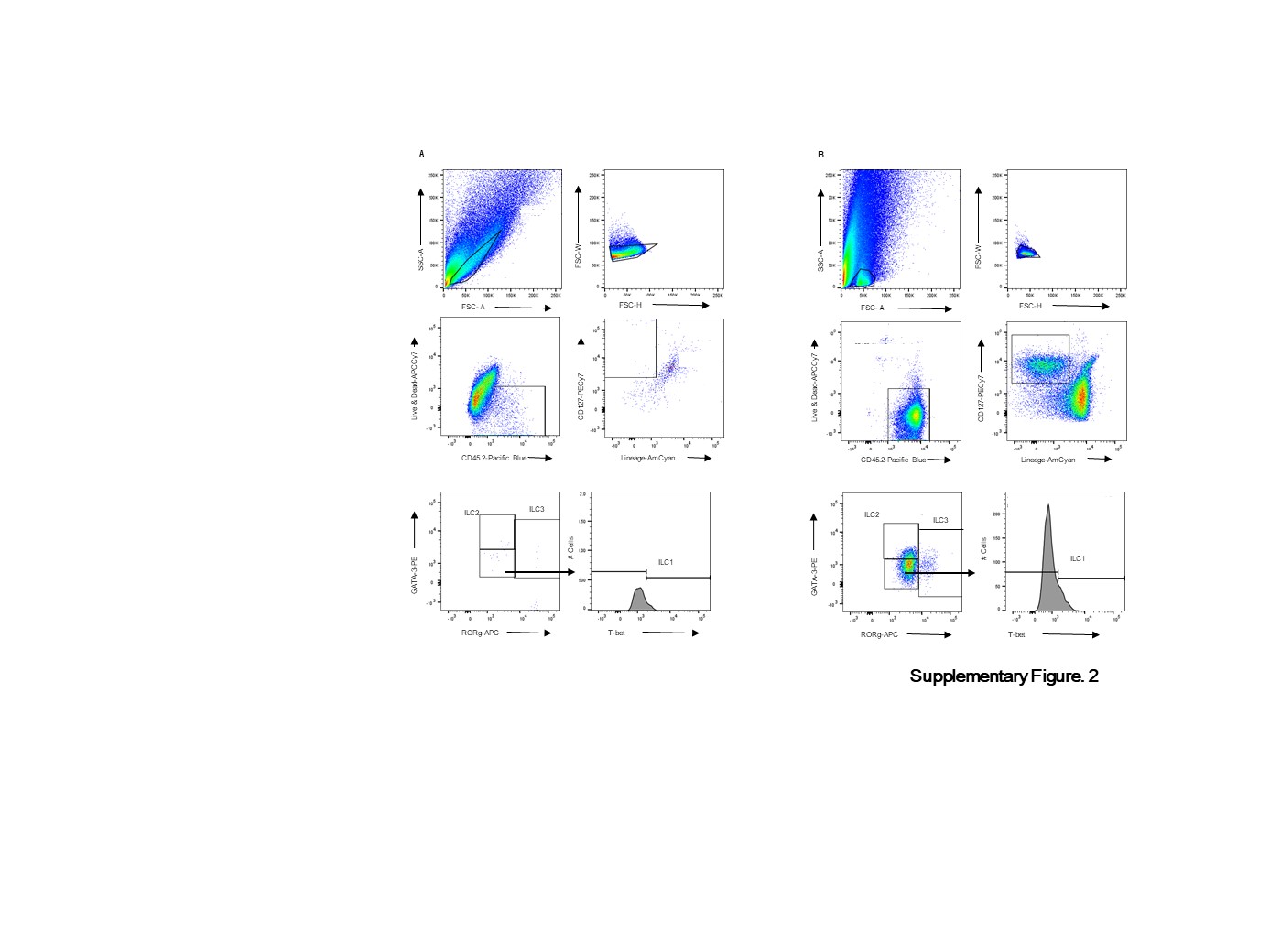


**Supplementary Figure 2.** **Gating strategy of innate lymphoid cells.**

Representative flow cytometry plots of ILC1s, ILC2s and ILC3s in the intestine (2A) and the liver (2B).

**
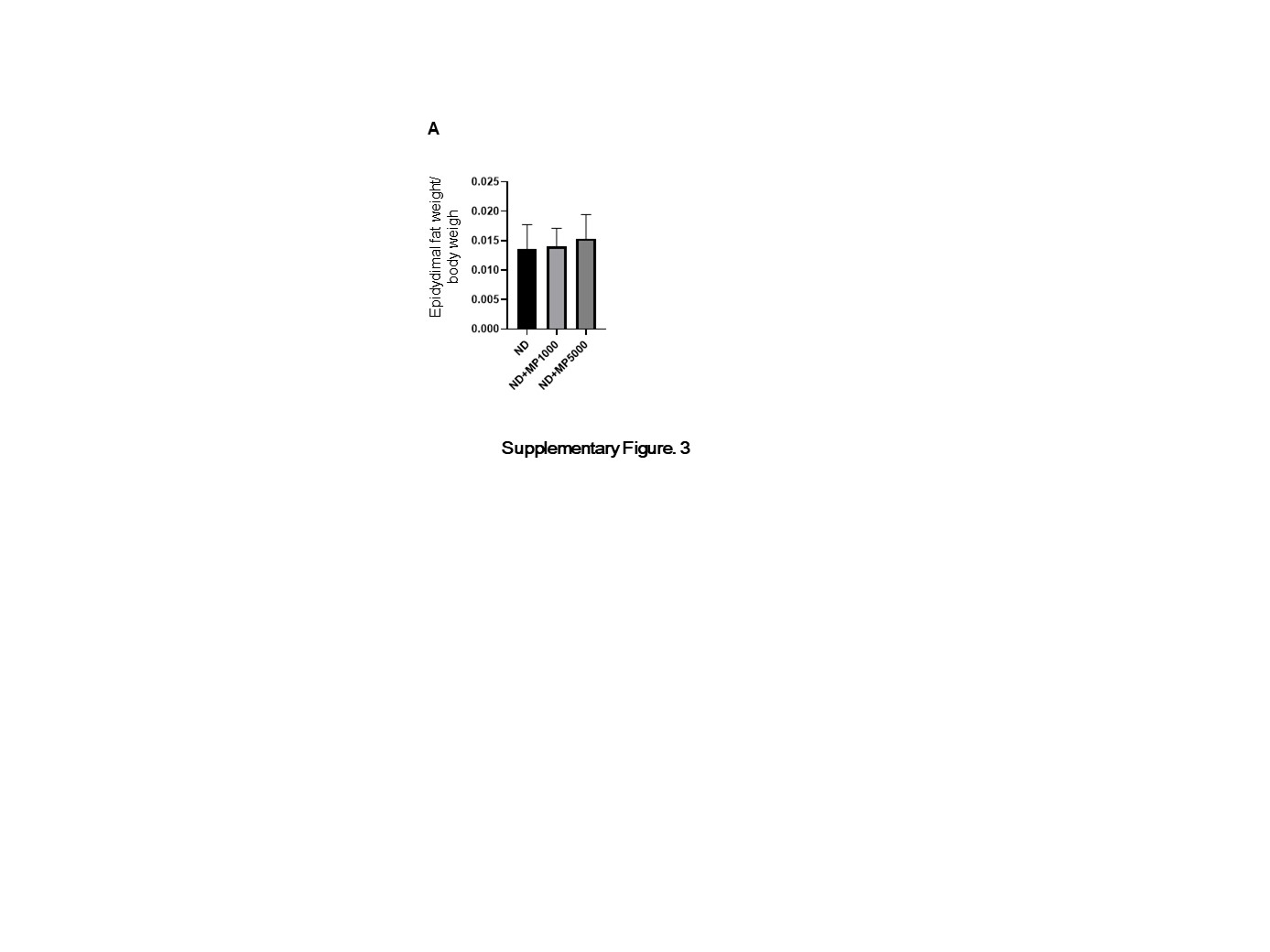
**

**Supplementary Figure 3. Relative epidydimal fat weight.**

Ratio of epidydimal fat weight to body weight. (n = 10).

**
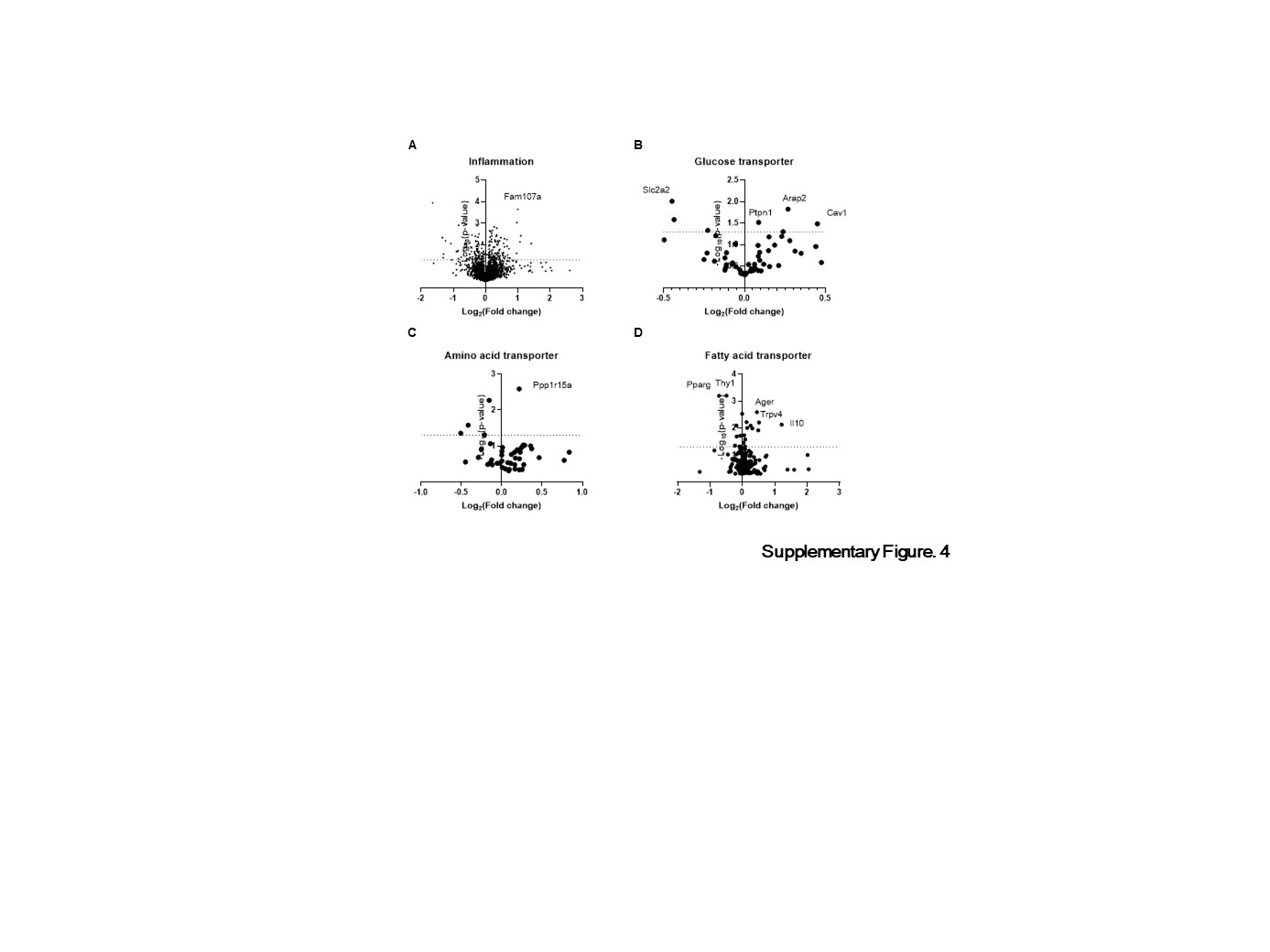
**

**Supplementary Figure 4. Changes in the expression of genes involved in intestinal inflammation and nutrient absorption (ND v.s. ND+MP 1000 µg/L mice).**

Global mRNA expression of gene related to **(A)** inflammation, **(B)** amino acid transporters, **(C)** glucose transporters, and **(D)** fatty acid transporters visualized as a volcano plot (n=3).
